# Supplementary material for: Circulating Small EVs miRNAs as Predictors of Pathological Response to Neo-Adjuvant Therapy in Breast Cancer Patients
Source: Int J Mol Sci. 2022 Oct 20;23(20):12625. doi: 10.3390/ijms232012625 (PMC9604084; doi:10.3390/ijms232012625)
Supplement: Supplementary file 1 [file ijms-23-12625-s001.zip › ijms-1944511-supplementary File S1.pdf]

Supplementary S1.

Our preliminary data pointed out that introducing a supplementary step for plasma filtration (0.8-µm filter), before isolation of the EVs by precipitation with Total Exosome Isolation Kit from plasma (Thermo Fisher), lead to improvement of the sEVs population by eliminating the large EVs.
EVs obtained by precipitation, with or without previous filtration were evaluated by TEM and nanoparticle tracking analysis (NTA). The analysis showed that larger particles were successfully eliminated by the filtration step, leading to the attaining of EVs with diameters in the range of 50-200 nm.

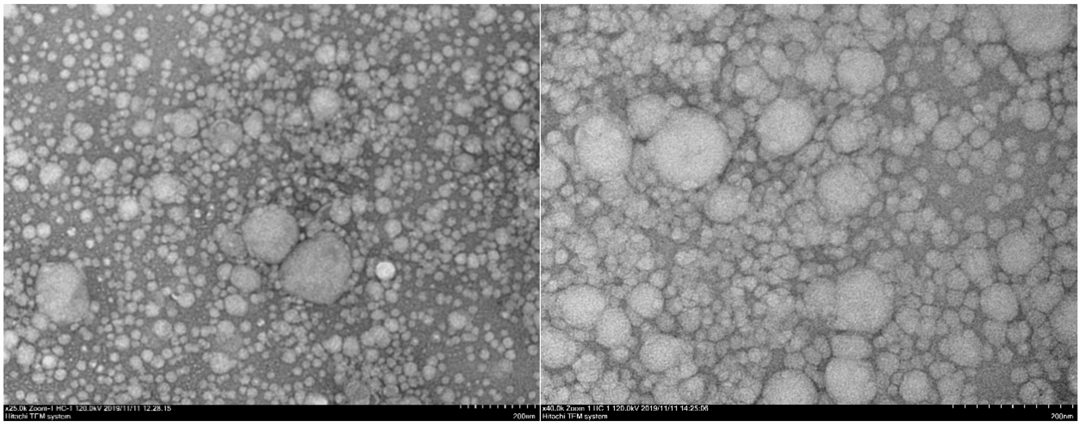

Figure S1. TEM images for Evs isolated from plasma **without** 0.8 µm filtration step.

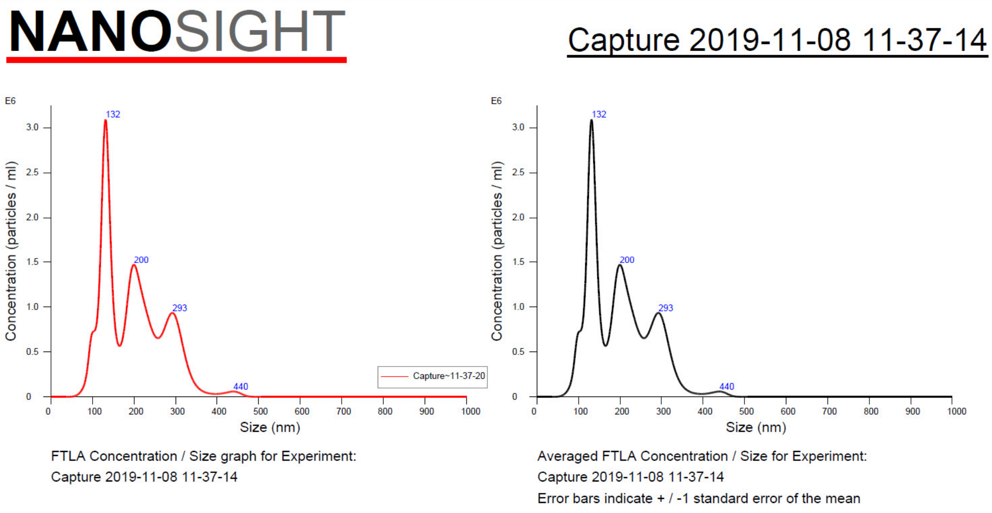

Figure S2. Nanoparticle tracking analysis (NTA), on EVs isolated from plasma **without** 0.8 µm filtration step.

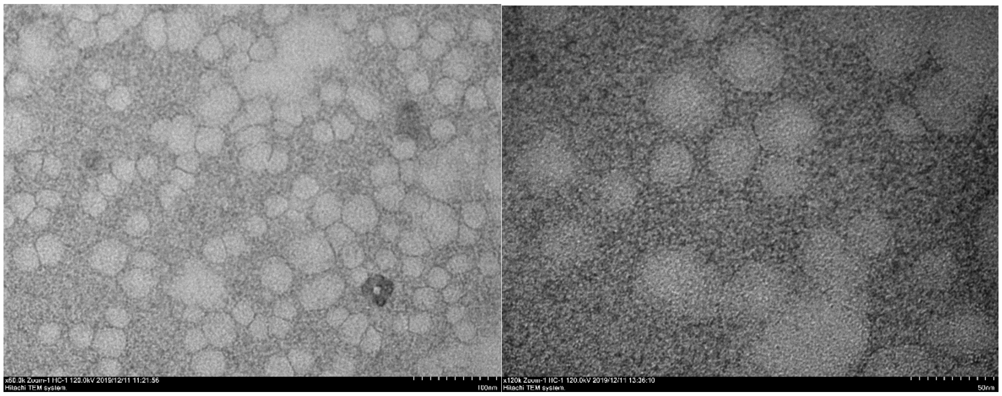

Figure S3. TEM images for Evs isolated from plasma **with** 0.8 µm filtration step.

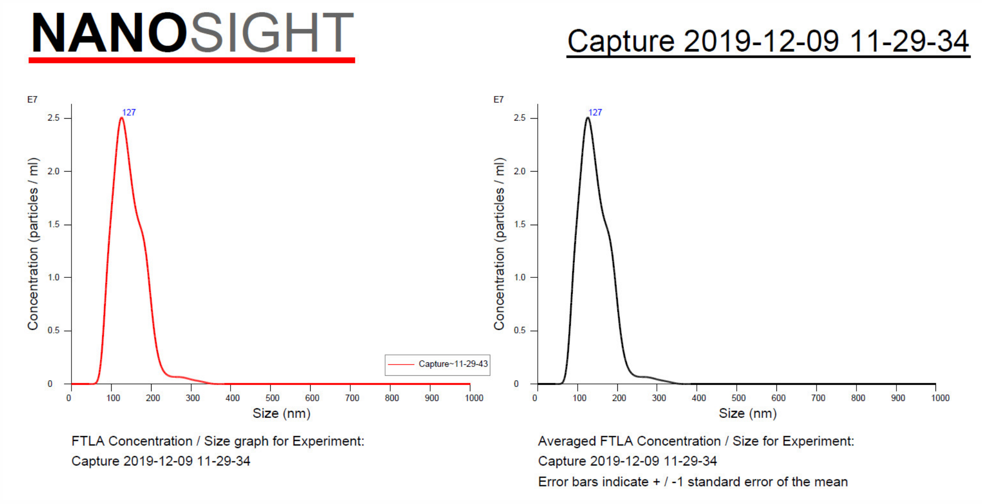

Figure S4. Nanoparticle tracking analysis (NTA), on EVs isolated from plasma **with** 0.8 µm filtration step.
